# Models and methods to characterise levonorgestrel release from intradermally administered contraceptives

**DOI:** 10.1007/s13346-021-01091-5

**Published:** 2021-12-03

**Authors:** Adnan Al Dalaty, Benedetta Gualeni, Sion A. Coulman, James C. Birchall

**Affiliations:** School of Pharmacy and Pharmaceutical Sciences, Cardiff University, Cardiff, CF10 3NB UK

**Keywords:** Levonorgestrel, Skin, Microneedles, Intradermal, Contraception, Method

## Abstract

Microneedle (MN)-based technologies have been proposed as a means to facilitate minimally invasive sustained delivery of long-acting hormonal contraceptives into the skin. Intradermal administration is a new route of delivery for these contraceptives and therefore no established laboratory methods or experimental models are available to predict dermal drug release and pharmacokinetics from candidate MN formulations. This study evaluates an in vitro release (IVR) medium and a medium supplemented with ex vivo human skin homogenate (SH) as potential laboratory models to investigate the dermal release characteristics of one such hormonal contraceptive that is being tested for MN delivery, levonorgestrel (LNG), and provides details of an accompanying novel two-step liquid–liquid drug extraction procedure and sensitive reversed-phase HPLC–UV assay. The extraction efficiency of LNG was 91.7 ± 3.06% from IVR medium and 84.6 ± 1.6% from the medium supplemented with SH. The HPLC–UV methodology had a limit of quantification of 0.005 µg/mL and linearity between 0.005 and 25 µg/mL. Extraction and detection methods for LNG were exemplified in both models using the well-characterised, commercially available sustained-release implant (Jadelle®). Sustained LNG release from the implant was detected in both media over 28 days. This study reports for the first time the use of biologically relevant release models and a rapid, reliable and sensitive methodology to determine release characteristics of LNG from intradermally administered long-acting drug delivery systems.

**Graphical abstract:**

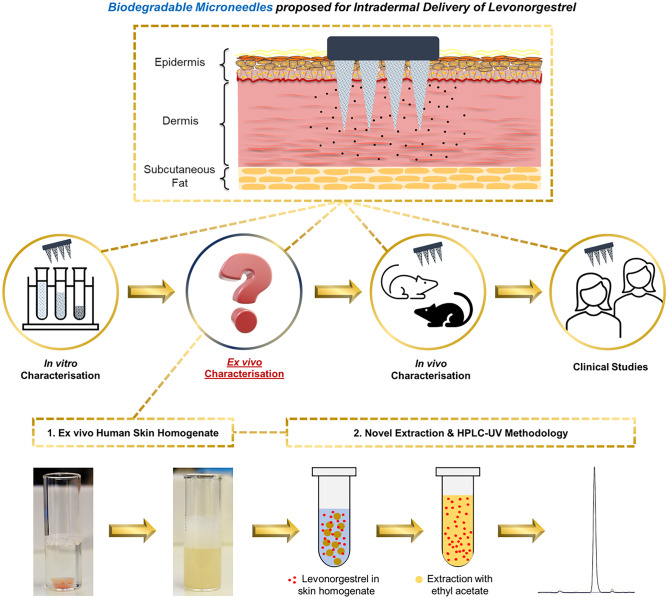

**Supplementary information:**

The online version contains supplementary material available at 10.1007/s13346-021-01091-5.

## Introduction

Providing women and adolescent girls with access to contraceptives and family planning services allows them to make their own decisions on whether they want to have children, when they want to have children and how many children they have [1]. Levonorgestrel (LNG), or d-Norgestrel, is a synthetic progestin commonly used for female contraception. LNG inhibits gonadotropin-releasing hormone release from the hypothalamus by activating progesterone and androgen receptors, resulting in inhibition of ovulation. It also increases the thickness of cervical mucus which obstructs the penetrability and survival of sperm [2]. LNG is the active pharmaceutical ingredient in contraceptive products that include emergency contraceptive pills [3], progestogen-only contraceptive pills [4], hormonal intrauterine devices [5] and subcutaneous implants [6]. Long-acting reversible contraceptives (LARCs) offer improved convenience and compliance and, through controlled daily release of the therapeutically-active dose, can minimise undesirable side effects [6, 7]. However, commercially available long-acting injections and subcutaneous implants require painful invasive administration, typically by trained healthcare professionals, generate biohazardous sharps waste and, in the case of implants, require removal within a clinical setting [7].

Microneedle (MN) technology [8–12] provides a potential platform for development of a minimally invasive alternative method of long-acting contraception [13, 14]. MNs are micron-sized projections designed to puncture the outermost layer of skin, the stratum corneum, to facilitate local delivery of the therapeutic cargo into the cutaneous space whilst minimising pain and bleeding [15]. Recent studies in female Sprague Dawley rats have evaluated systemic uptake of LNG [16–19] or etonogestrel [20] following intradermal delivery of these progestin-only contraceptives from biodegradable MN systems. Enzyme-linked immunosorbent assay (ELISA) [17, 18] or HPLC/UPLC-MS [16, 19, 20] was used to analyse LNG levels in plasma; however whilst these in vivo studies in small mammals provide valuable data on systemic uptake of MN administered progestins, they do not fully characterise the local behaviour of the implant, i.e. drug release in the skin, and the results are caveated by acknowledged differences in the architecture and biology of human and rodent skin [21, 22]. In vivo studies are also time-consuming, expensive and subject to ethical scrutiny, and therefore, in vitro models and methods to better understand sustained intradermal progestin delivery are highly desirable.

The most prevalent apparatus used for in vitro laboratory characterisation of cutaneous dosage forms is static (Franz-type) and flow-through (Bronaugh-type) diffusion cells [23]. However, these tools are principally designed to characterise permeation of active pharmaceutical ingredients across a rate-limiting membrane that is designed to imitate the skin barrier. For a dosage form that negates the biological skin barrier, i.e. an intradermal implant, the critical attribute that needs to be characterised is drug release within the skin environment. In vitro dissolution tests for transdermal drug delivery systems [24] have therefore been adopted and adapted to characterise drug release from biodegradable MN devices [17–20, 25]. However, whilst these studies help characterise the dosage form, their biological relevance and correlation to the in vivo performance of an intradermal implant is unknown. Additional pre-clinical models and methods are therefore required to rapidly evaluate the delivery and, in the case of long-acting methods, the release characteristics of MN administered contraceptives in the dermal space.

Laboratory models must also be accompanied by a robust but sensitive validated analytical method that can detect and quantify candidate drugs, such as LNG, at clinically relevant concentrations. A range of analytical techniques including radioimmunoassay (RIA) [26, 27], ELISA [17, 18, 28], gas chromatography with mass spectrometry (GC–MS) [29, 30], liquid chromatography with mass spectrometry (LC–MS) [31–35] and high performance liquid chromatography (HPLC) accompanied by either ultraviolet (UV) [36, 37] or photo-diode array [38, 39] detection have been successfully used to quantify LNG in human plasma [27, 29, 32, 33, 35], human serum [31, 34], breast milk [26, 30], human and primates’ urine [28], rat plasma [17, 18, 36] and in vitro media [37–39]. Many of these methodologies also rely upon an extraction step, typically performed using liquid–liquid extraction (LLE) [32, 33, 35]; however, the use of protein precipitation (PPT) [33, 34, 40] and solid phase extraction (SPE) [41] has also been explored. However, there are no published studies describing the extraction and quantification of LNG in skin or skin models.

The aim of this study is to develop accessible, rapid, sensitive and reliable laboratory methods that can accurately determine intradermal release from sustained-release LNG dosage forms, using an in vitro release (IVR) medium and an ex vivo human skin homogenate (SH) medium accompanied by validated optimised extraction and analytical methods. Whilst this research has wide applicability to those developing intra- or trans-dermal contraceptive delivery systems, it is of particular relevance to current international efforts to develop MN-mediated progestin-based LARCs [13, 14].

## Materials and methods

### Materials

Lyophilised LNG (pharmaceutical grade of European Pharmacopeia reference; 99.9% pure; MW: 312.45 g/mol), sodium phosphate monobasic and sodium phosphate dibasic were obtained from Sigma-Aldrich, UK. Jadelle® subcutaneous implants (Bayer, Germany) were a kind gift from the Population Council, New York, USA. LC–MS-grade water and acetonitrile, and hexane, isoamyl alcohol, ethyl acetate, diethyl ether, tert-butyl methyl ether and chloroform were purchased from Fisher Scientific, UK, and used as received. Sodium dodecyl sulphate (SDS) was purchased from VWR, UK. Phosphate-buffered saline (PBS) pH 7.2 was from Gibco (Thermo Fisher, UK). De-ionised water was obtained from a Purite water purification system, USA. All other solvents and chemicals were of analytical grade and stored at room temperature. A Techne sample concentrator supplied with a stream of gas nitrogen was used for the evaporation of organic solvents in a dry bath (Labnet digital, USA). The UV–Vis spectrophotometer was a Cary 60 (Agilent Technologies UK Ltd). Microcentrifuge tubes were vortex-mixed using an infrared vortex mixer (F202A0175FI, Fisherbrand, UK) and centrifuged using a microcentrifuge (Heraeus Fresco 17, Thermo Scientific, UK).

#### HPLC–UV instrument

Reversed-phase (RP) HPLC–UV analysis was performed using an UltiMate-3000 system (Thermo-Fisher, UK). The system is composed of an UltiMate LPG-3400SD standard quaternary pump, an UltiMate WPS-3000SL/TSL standard well plate autosampler, an UltiMate 3000 TCC-3000SD standard thermostatted column compartment and an UltiMate 3000 VWD variable wavelength detector. Chromatographic separation was performed using a C18 Kinetex® RP-HPLC column (4.6 × 250 mm, particle size 5 µm, 100 Å), Phenomenex, USA. The column was protected by a SecurityGuard ultra cartridge (4.6 mm i.d.), Phenomenex, USA. Instrumental control of the HPLC system as well as data collection, processing and integration used Chromeleon 7.2 SR4 software (Thermo-Fisher, UK).

#### Human breast skin explants

Skin explants were collected following breast reduction surgery or mastectomy at the Royal Gwent Hospital, Newport, UK. The explants were obtained under informed written patient consent and local ethical committee approval (LREC Ref: 08/WSE03/55). Excised skin was transported to the laboratory in Dulbecco’s modified Eagle’s medium (DMEM) supplemented with 2% penicillin and streptomycin (10,000 IU/mL) and then used immediately, or frozen (−20 °C) and used at a subsequent time, as previously described [42].

### Development of a HPLC–UV method to quantify LNG

#### Preparation of LNG calibration standards for HPLC–UV

LNG powder was transferred to a 2 mL microcentrifuge tube and its mass indirectly determined using a micro-balance (Sartorius MC 5, Germany). A standard LNG stock solution (360 µg/mL) was prepared using LC–MS grade acetonitrile (ACN). LNG calibration standards: 25, 15, 5, 1, 0.5, 0.25, 0.05, 0.01, 0.005, 0.0025 and 0.001 µg/mL were then prepared by serial dilution with LC–MS ACN to create two calibration curves, 0.005 to 0.5 µg/mL and 0.5 to 25 µg/mL. LNG calibration standards were also used to ‘spike’ the IVR (0.4, 1.6, 3.6 and 19.0 µg/mL) and SH (3.6 and 19.0 µg/mL) media to evaluate method specificity. Standard LNG stock solutions, as well as calibration standards (stored in 2 mL glass HPLC vials), were stored at – 20 °C. All samples were equilibrated in the HPLC sampler at 4 °C for 15 min prior to use.

#### HPLC–UV method development and optimisation

Initial HPLC–UV analyses were performed using 20 µL of the LNG calibration standard, 15 µg/mL, and an isocratic mobile phase containing LC–MS grade ACN/water (ACN:H_2_O) 80:20 (v/v) with a flow rate of 1 mL/min for a total run time of 12 min. The column temperature was set at 25 °C, and the UV absorbance was recorded at 236 nm. The method was then optimised by independent modification of (i) mobile phase composition, (ii) flow rate, (iii) the wavelength for UV detection and (iv) column temperature. Isocratic and gradient elution methods were examined, and several mobile phase ratios (ACN:H_2_O) were tested between 50:50 and 100:00 (v/v). The wavelength for detection was optimised within the UV range of 225 to 248 nm. The flow rates examined included 0.5, 0.75, 1 and 1.25 mL/min. Column temperatures between 25 and 50 °C were also evaluated within this optimisation process.

#### Optimised HPLC–UV method

The optimal chromatographic conditions for LNG detection consisted of a gradient elution of ACN:H_2_O, at a flow rate of 1 mL/min, starting at 60:40 (v/v), changing to 100:00 (v/v) at 4.4 min and then returning to 60:40 (v/v) at 5 min. The injected sample volume was 20 µL, and LNG absorbance was recorded at 244 nm. The column temperature was set at 37 °C, and a single run lasted 7.5 min. Negative controls for HPLC–UV analyses included LC–MS grade ACN and IVR medium.

### Validation of the optimised HPLC–UV method for LNG

The optimised HPLC–UV method was validated using LNG calibration standards according to recommendations of the International Council for Harmonisation (ICH) [43] and European Pharmacopeia [44]. The method was validated in terms of specificity, linearity, sensitivity, precision and accuracy.

#### Specificity

The assay was developed to quantify LNG in both IVR and SH media (see “Preparation of in vitro release medium and ex vivo human skin homogenate medium to analyse LNG release”). The HPLC–UV chromatograms of (i) negative controls of ACN, IVR medium and SH medium; (ii) LNG calibration standards (7.5 and 15 µg/mL) in ACN and (iii) spiked LNG preparations in IVR medium or SH medium were recorded using the optimised HPLC–UV method (“Optimised HPLC–UV method”). Specificity was evaluated by visual inspection of the recorded chromatograms and quantitative comparison of the area under the curves (AUCs) and retention times (RT) of the LNG peaks. Samples containing LNG in ACN or IVR medium were analysed directly, while LNG in SH medium was subjected to an extraction step (“LLE of LNG from human skin homogenate medium”) prior to HPLC–UV analysis.

#### Linearity

Nine LNG calibration standards were prepared in triplicate, assayed using the optimised HPLC–UV method and analysed (by calculating AUCs) to construct two calibration curves, with each curve containing 5 concentrations (0.005 to 0.5 µg/mL and 0.5 to 25 µg/mL). The ‘least squares’ method was used to assess linearity.

#### Sensitivity

Method sensitivity was evaluated by determining the limits of detection (LOD) and quantification (LOQ). LOD and LOQ were estimated using the ‘signal-to-noise (S/N)’ method specified in the European Pharmacopeia [44]. The peak-to-peak noise in proximity to the LNG peak was measured, and LOD and LOQ were estimated to be 3 and 10 times greater than the S/N value, respectively, according to the formula: S/N = 2H/h, where *H* is the height of LNG peak and *h* is the maximum peak-to-peak noise within a time period equating to 20 times the peak width at half-height. Three independent experiments (*n* = 3) were used to calculate mean LOD and LOQ values.

#### Precision

Intra-day (repeatability) and inter-day (intermediate) precisions were evaluated by analysing LNG calibration standards (“Linearity”) (i) on the same day (*n* = 3) and (ii) over three consecutive days (*n* = 3) respectively. Relative standard deviations (%RSD) and relative errors (%RE), expressed as a percentage, were calculated.

#### Accuracy

Method accuracy was evaluated in accordance with the ‘standard additions’ method whereby a known volume of LNG calibration standard (0.5, 1 and 5 µg/mL) was added to a previously defined sample (1 µg/mL), to achieve either 25%, 50%, 100% or 200% additions to the concentration. ICH guidance for accuracy validation recommends conducting a minimum of nine determinations over a minimum of 3 concentrations of the analyte [43]. The accuracy was then calculated by comparing the theoretical added mass of LNG to the measured value. Measurements were performed in triplicate and the %RSD and %RE were calculated.

### Preparation of in vitro release medium and ex vivo human skin homogenate medium to analyse LNG release

IVR medium consists of 100 mM phosphate buffer supplemented with 0.5% sodium dodecyl sulphate (SDS) and is adjusted to pH 7.4 using 1 M sodium hydroxide.

To prepare SH medium, frozen excised human breast skin explants (“Human breast skin explants”) were first defrosted and subcutaneous fat removed by blunt dissection. The skin was then rinsed in PBS 1 × (10 mM, pH 7.2), secured on foil-covered dissection corkboards (epidermis side up) and surface wiped with 70% v/v ethanol prior to removal of biopsies (8 mm diameter) using a biopsy punch (Miltex, Japan). Biopsies were immersed in PBS (1 ×) before transfer to a glass tube containing 2 mL of IVR medium. Biopsy homogenisation was achieved using a vertical bar homogeniser (Polytron PT 1600E, Kinematica, Switzerland) at progressively increasing speeds (1000–30,000 rpm) until a visibly uniform suspension was obtained. Homogenates of several skin biopsies, from the same donor, were pooled, and 2 mL of this pooled homogenate was then further diluted with IVR medium to a final volume of 7.5 mL. The resulting medium is termed SH medium.

### Development of a liquid–liquid extraction (LLE) procedure to isolate LNG from human skin homogenate medium

#### LLE of LNG from human skin homogenate medium

**Fig. 1:**
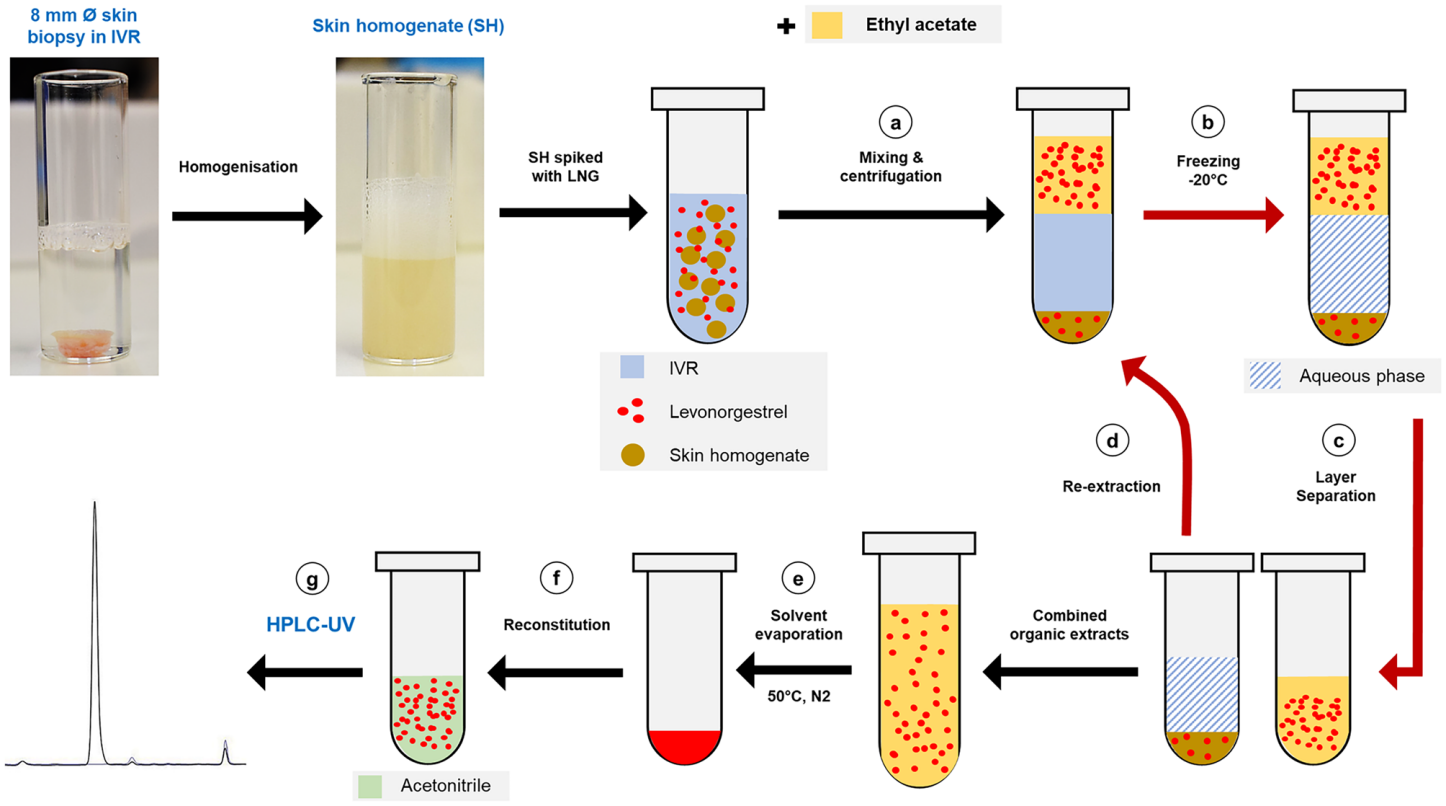
Schematic illustration of the validated LLE (liquid–liquid extraction) procedure for LNG extraction from SH medium. SH medium was prepared by homogenising an 8 mm diameter skin biopsy in IVR medium using a vertical bar homogeniser. The SH medium was then spiked with an LNG calibration standard and extracted with ethyl acetate as follows: the mixture was vigorously vortex-mixed and then centrifuged (**a**). The mixture was then stored at –20 °C (**b**) allowing the aqueous phase to freeze and enabling the organic supernatant to be collected (**c**). The aqueous layer was then thawed and re-extracted (**d**), and the combined organic extracts were evaporated to dryness (**e**). Finally, the residual powder was reconstituted in acetonitrile (**f**) and analysed by HPLC–UV (**g**)

The extraction procedure was developed in three stages. In Stage 1 (Solvent selection), LNG was spiked into both water and IVR medium, and a range of organic solvents (Table 1), identified from previous published (Table 1) and unpublished work, were evaluated for their ability to recover the spiked LNG. In this preliminary stage, the extraction process (described in detail in Fig. 1) used a single extraction step (i.e. re-extraction identified as step d in Fig. 1 was omitted) due to the simplicity of the aqueous medium. To perform the extraction, 1 mL of the organic solvent was added to 1 mL of the LNG spiked media, and extractions were performed in triplicate. The process was repeated using both water and IVR medium and using two different concentrations of LNG, nominally referred to as ‘high’ (1.6 µg/mL) and ‘low’ concentrations (0.4 µg/mL).

**Table 1:** Initial LLE (liquid–liquid extraction) procedures were conducted using a range of solvents. A list of the organic phases that were evaluated and the reference source used to identify them are presented in this table

**Organic solvents**	**Ratio**	**Reference**
Hexane: iso-amyl alcohol	90:10	[36]
Hexane: iso-amyl alcohol	98:02	[32]
Hexane/ethyl acetate	70:30	[40]
Di-ethyl ether	100	[26, 31]
Tert-butyl methyl ether	100	[35]
Ethyl acetate	100	[33]
Chloroform/methanol	2:1	–
Hexane	100	–
Iso-amyl alcohol	100	–

Selection of the most appropriate organic phase for extraction (Stage 1) was followed by a two-step LNG extraction procedure (Fig. 1) to try and increase the extraction efficiency from the more complex biological SH medium. Stage 2 (Two-step extraction) was evaluated using both IVR medium and SH medium (*n* = 3 each), each spiked with LNG to produce 950 µL medium containing 19.0 µg/mL LNG. The extractions used 500 µL of ethyl acetate in the first extraction step and 150 µL of ethyl acetate in the re-extraction step.

Stage 3 (Optimisation and validation) involved minor refinements to the protocol and examined the reproducibility of the extraction process in SH medium over 3 consecutive days (performed in triplicate on each day). In this final stage, SH samples (*n* = 9) were spiked with LNG to produce a 1 mL spiked medium containing 3.6 µg/mL LNG. The optimised two-step extraction procedure, illustrated for the SH medium in Fig. 1, was then used to recover the LNG. Briefly, 550 µL of ethyl acetate was added to 500 µL of SH sample in a 2 mL microcentrifuge tube. After equilibration at room temperature for at least 15 min, the mixture was vortexed at 2400 rpm for 3 min and then centrifuged for 5 min at 4000 rpm, (step a). The aqueous phase was then frozen at –20 °C, to separate the organic and aqueous layers (step b), and the organic supernatant was manually extracted using a pipette (step c). The thawed aqueous residue was then re-extracted with 165 µL of ethyl acetate following the same procedure (step d). The extracts were then combined and evaporated to dryness in a sample concentrator, under a stream of nitrogen gas at 50 °C (step e). The residual powder was then reconstituted in 200 µL of ACN, vortex-mixed for 1 min at 2400 rpm (step f) and finally analysed using an optimised HPLC–UV method to quantify LNG (step g).

#### Calculation of extraction recovery efficiency

Two extraction controls (referred to as *EC1* and *EC2*) were prepared by spiking ACN with known volumes of an LNG calibration standard. *EC1* was a positive control that was not subjected to any of the extraction protocol (“LLE of LNG from human skin homogenate medium”). *EC2* was a positive control subjected to the evaporation and reconstitution steps (Fig. 1, steps e–f) to determine if evaporation resulted in loss of the LNG analyte. Both ECs were analysed using the optimised HPLC–UV method. The extraction recovery efficiencies of LNG were calculated in comparison to *EC1* according to the formula:

$$\mathrm E\mathrm x\mathrm t\mathrm r\mathrm a\mathrm c\mathrm t\mathrm i\mathrm o\mathrm n\;\mathrm R\mathrm e\mathrm c\mathrm o\mathrm v\mathrm e\mathrm r\mathrm y\;\mathrm E\mathrm f\mathrm f\mathrm i\mathrm c\mathrm i\mathrm e\mathrm n\mathrm c\mathrm y\%=\frac{{Con}_{\mathrm{sample}}\times V_{\mathrm{sample}}\times{DF}_{\mathrm{sample}}}{{Con}_{\mathrm{EC}1}\times V_{\mathrm{EC}1}\times{DF}_{\mathrm{EC}1}}\times100$$

where:

- *Con*_sample_ and *Con*_EC1_ (µg/mL) are the measured LNG concentrations within the extracted sample and EC1 respectively.
- *V*_sample_ and *V*_EC1_ (mL) are the volumes of the extracted sample and EC1 control.
- *DF*_sample_ and *DF*_EC1_ are the dilution factors of the extracted sample and EC1 before the HPLC–UV analysis.

### Evaluation of in vitro release medium and ex vivo human skin homogenate medium to characterise LNG release from a commercially available sustained release formulation

A commercially available subcutaneous implant, Jadelle® (75 mg LNG per rod), was used to confirm the suitability of the models and method developed in this study. LNG release with time was characterised by placing a single Jadelle® rod in 15 mL of IVR medium or SH medium, in a shaking (200 rpm) water bath maintained at 37 °C. At 7, 14 and 28 days, a 500 µL sample was removed from the release medium (which was replenished with fresh medium) for analysis. IVR and SH samples were extracted with ethyl acetate according to the optimised protocol (Fig. 1), and LNG was quantified using the optimised HPLC–UV method (“Optimised HPLC–UV method”). A further sample was taken from the IVR medium and directly analysed, i.e. without being subjected to the extraction process, using the optimised HPLC–UV assay.

## Results and discussion

Microneedles have emerged in recent years as a potential platform for sustained release of LNG, and other contraceptives, into the skin. This is an exciting, internationally recognised clinical application for microneedles, as exemplified by a recent review and a business case analysis commissioned by PATH [13, 14]. There are however no standardised in vitro and ex vivo models or methods to examine sustained progestin release in the skin because there are no established methods of drug delivery that can target this body compartment. The emergence of microneedle-based progestin delivery systems therefore creates a need to develop validated laboratory tools. These tools must be skin specific (as skin is the target organ for microneedles) and highly sensitive (because LNG release from microneedles is likely to be in the low microgram range). This study introduces new biologically relevant in vitro/ex vivo drug release models, an optimised extraction procedure and a validated sensitive HPLC–UV methodology which, when used in combination, can accurately evaluate and understand LNG release kinetics in the dermis at ng/mL concentrations.

### Developing supplemented media to evaluate LNG release in the skin

The development of MN approaches that confer extended drug release challenges the boundaries of existing laboratory models. Whereas existing commercial LARC products deliver the progestin cargo into subcutaneous (implant) or intramuscular (injection) compartments, MN-based approaches deliver the drug into the skin. Whilst it would be preferable to monitor the drug release profile within intact excised skin, this is only possible over a few days, rather than several months. An initial evaluation of drug release and dissolution can however be performed using relatively simple apparatus and IVR media adapted (with respect to composition, pH, volume, temperature) for the specific analyte and formulation. Several IVR media have been successfully used to characterise LNG or etonogestrel release from MNs and other delivery systems. Examples include standard PBS [25, 45] or PBS supplemented with a surfactant, such as 0.5% SDS [25] or polyethylene glycol 400 at 30% [20] or 40% [19], to facilitate dissolution of the lipophilic analytes. Accelerated release conditions can also be created by addition of an organic solvent such as 25% ethanol [45, 46] or an organic solvent (e.g. ethanol, isopropanol, tert-butanol or tetrahydrofuran) with a surfactant (Tween-20, Tween-80 or SDS) [17, 18, 45, 47]. Whilst such studies allow for an abbreviated, yet informative, release study, the influence of the solvent conditions on the release mechanism and kinetics should be viewed with caution when interpreting the data. This study used a phosphate-buffered medium supplemented with 0.5% SDS to provide a simple, yet valuable, IVR model for comparing LNG release from different formulations at the early screening stage.

However, drug release within a simple aqueous environment may not be predictive of drug release within the skin. Previous studies have investigated the diffusion and stability of molecules within skin matrices. Ito et al. [48] evaluated the stability of leuprorelin acetate delivered to skin via MNs using a rat SH model [48]. Other groups have used SH of animal origin as surrogates for in vivo conditions to study the impact of exogenous fatty acids on bacterial membrane composition and function [49], to measure the concentrations of different metabolites [50–52] and to evaluate the accuracy of theranostic applications [53].

In this study, we developed and tested a representative ex vivo drug release model containing all of the biological components of human skin, i.e. IVR medium supplemented with human SH, to provide a bridge between in vitro and in vivo drug release studies.

### Development, optimisation and validation of a sensitive HPLC–UV method to quantify LNG

A reliable method to detect potent drug molecules released from an intradermally administered long-acting contraceptive will require high levels of sensitivity, specificity and repeatability. In this study, a HPLC–UV chromatographic methodology was iteratively developed (summarised in Supplementary Table 1) to optimise peak sharpness, AUC, retention time and separation. A simple LNG solution was initially analysed adapting a published method [36, 38], and this produced a sharp symmetrical LNG peak at a retention time of 3.65 min, with neither a peak front, tail or interfering peaks compared to the negative control. However, quantification of LNG within a biologically representative medium, i.e. SH, necessitated a drug extraction step (“LLE of LNG from human skin homogenate medium”) and optimisation of the HPLC–UV assay to eliminate the impact of potential interferent on LNG quantification.

**Fig. 2:**
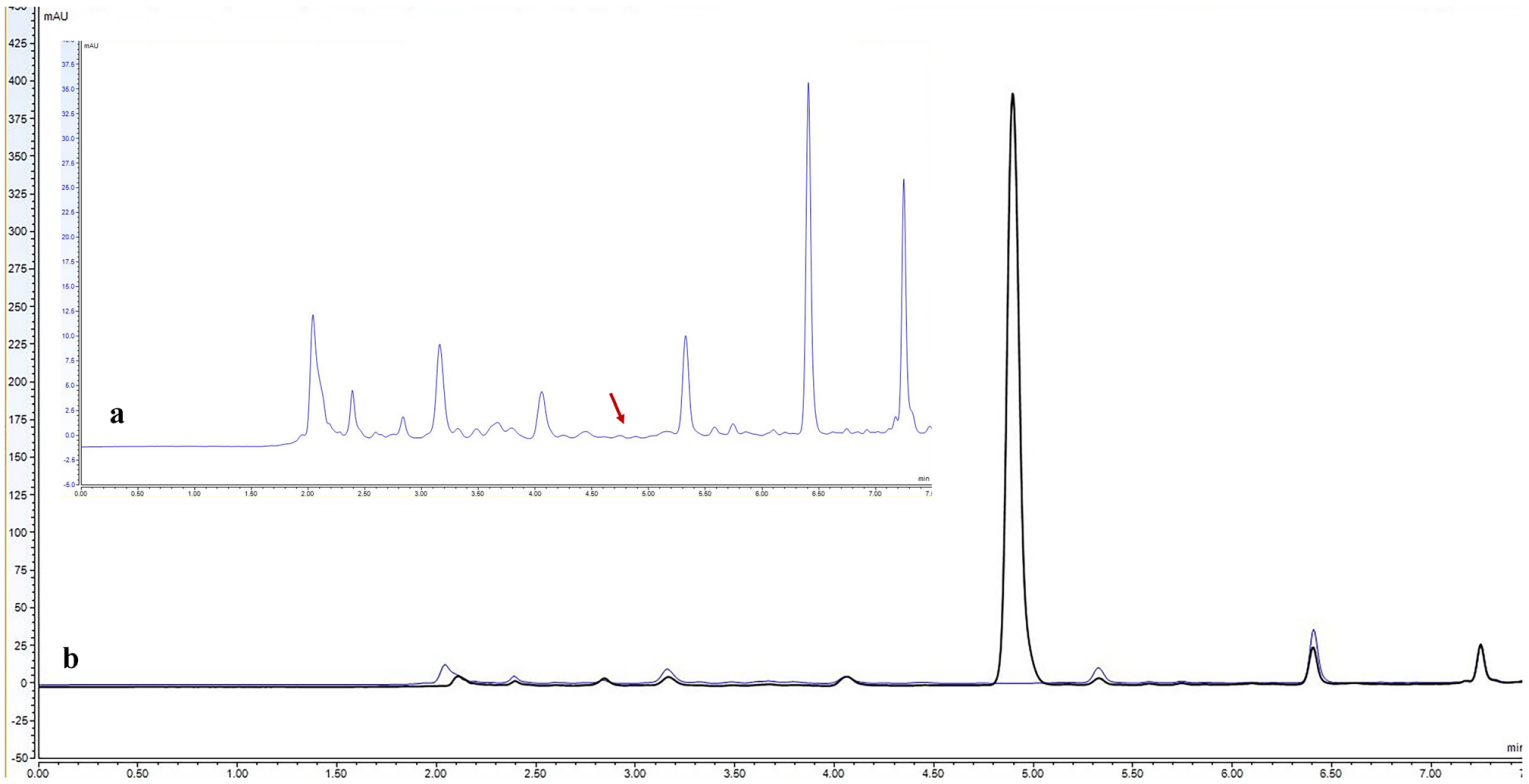
Illustrative overlaid HPLC–UV chromatograms of (**a**) the extract of SH medium and (**b**) the extract of LNG spiked in SH medium. (**a**) Magnified HPLC–UV chromatogram of extracted SH medium (negative control), with the red arrow indicating the absence of interfering peaks at the optimized LNG retention time. (**b**) Representative HPLC–UV chromatogram of extracted LNG (black) from spiked SH medium (1.6 µg/ml) compared to the SH extract alone, i.e. the negative control (grey). A sharp, symmetrical peak at ~ 4.8 min is LNG. Both SH extracts were prepared while developing the two-step extraction procedure and analysed using the optimised HPLC–UV method (“Optimised HPLC–UV method”). X axis: time (min), Y axis: UV absorbance (mAU)

Separation efficiency was enhanced by progressively reducing the proportion of ACN in the ACN:H_2_O mobile phase from 100% to 50%. This increased the retention time of the peak from 3.22 to 9.56 min (LNG logP = 3.8), a strategy that was also used by Zaidi et al. [37] for LNG quantification. However, an increased retention time was associated with a concomitant reduction in peak sharpness, an undesirable characteristic that reduces the sensitivity. A gradient elution mode was therefore employed, initiated at a mobile phase ratio of 60:40%v/v (ACN:H_2_O) and changed to either 90:10, 95:05 or 100:00%v/v at 4.4 min to facilitate more rapid elution of LNG through the column, before returning to 60:40%v/v. Each of the ratios tested in this gradient elution method provided sharp symmetrical peaks. However, the key factor was to ensure that the LNG peak was distinct from potential interferent in the extracted SH (negative controls) (Fig. 2a). An ACN:H_2_O ratio of 100:00 (v/v) at 4.4 min to 5 min was therefore selected as the preferred mobile phase as it produced an LNG retention time (~ 4.8 min) that was chronologically distinct from significant interferent peaks (Fig. 2b). Whilst gradient elution is not commonly used in the published literature to detect LNG, Wang et al. [41] and Cirrincione et al. [35] used this approach to detect LNG within human plasma samples following administration via oral [41] and subdermal implants [35]. The data reported in this study therefore indicates that gradient elution can also facilitate HPLC–UV analysis of LNG extracted from human skin.

Optimisation of the mobile phase composition was accompanied by refinement of method parameters (detailed in Supplementary Table 1) to further increase sensitivity, including the flow rate (1.0 mL/min) [54], the wavelength for UV absorbance of LNG (244 nm) and the column temperature (37 °C). Using these optimised conditions, the tailing factor of the LNG peak was < 2 in ACN, IVR and extracted SH, which fulfils the recommendations of the FDA for peak symmetry [55]. The principal characteristics of this optimised HPLC–UV assay were then evaluated in turn, as described in “Specificity,” “Linearity and sensitivity,” “Precision,” to “Accuracy.”

#### Specificity

Sharp symmetrical LNG peaks were recorded at ~ 4.8 min in ACN and IVR (Supplementary Fig. 1) at a range of LNG concentrations 0.005 to 25 µg/mL. The retention time and peak shape are comparable to those observed in the SH extract (Fig. 2b) and thus demonstrate that major cutaneous interferent, which are eluted both before and after the LNG peak, do not have a significant impact on the specificity of the method. A similar approach was previously reported by Liu et al. [33].

#### Linearity and sensitivity

Assay linearity was established over a wide range of concentrations, with two calibration curves (0.005 to 0.5 µg/mL and 0.5 to 25 µg/mL) producing correlation coefficients equal to 1 (Supplementary Fig. 2). The LOD and LOQ for LNG using the optimised HPLC–UV method was 0.001 µg/mL and 0.005 µg/mL respectively (Supplementary Table 2), which is a significant improvement on values that are typically reported in the published literature for HPLC–UV methods, e.g. 2.79 µg/mL [39] and 10 µg/mL [56], and is equivalent to the LOQ (5 ng/mL) [31] achieved using LC-Tandem MS to quantify LNG extracted from human serum samples. Whilst lower LOQ values are possible for LNG, e.g. 0.265 ng/mL [32], < 50 pg/mL [26] and 2.2 pg/mL [57], these rely on detection methods that are more sensitive than UV–Vis spectroscopy, namely mass spectrometry, radioimmunoassay or enzyme-immunoassay. Most importantly, the level of sensitivity achieved using the optimised HPLC–UV method is predicted to be sufficient for determining clinically relevant concentrations of LNG that will be released from sustained release formulations in the IVR and SH models.

#### Precision

The intra- and inter-day precisions of the HPLC–UV method were evaluated by determining the relative standard deviation (%RSD) and relative error (%RE) of nine calibration standard concentrations (*n* = 3) over three consecutive days (Table 2). Intra-day precision of the method produced %RSD values between 0.26% and 1.4%, while for the inter-day precision, %RSD values ranged from 0.08 to 4.16% (Table 2). As might be expected, relative errors were greatest as values approached the LOQ (0.005 µg/mL).

**Table 2:** Intra- and inter-day precisions of the HPLC–UV method. Each LNG calibration standard was analysed three times on the same day (intra-day precision), or once over 3 days, (inter-day precision). Data is presented as a mean concentration ± standard deviation (SD), the percentage RSD (relative standard deviation) and percentage RE (relative error)

Theoretical concentration (µg/mL)	**Intra-day precision**			**Inter-day precision**		
	Measured concentration ± SD (µg/mL)	± RSD (%)	± RE (%)	Measured concentration ± SD (µg/mL)	± RSD (%)	± RE (%)
0.005	0.0053 ± 0.000018	0.34	7.85	0.0053 ± 0.0002	4.16	6.37
0.01	0.01 ± 0.0001	1.09	0.67	0.01 ± 0.00008	0.8	0.46
0.05	0.0497 ± 0.0006	1.4	0.4	0.0497 ± 0.0002	0.53	0.42
0.25	0.25 ± 0.002	1.11	0.18	0.249 ± 0.0009	0.36	0.2
0.5	0.503 ± 0.005	1.12	0.77	0.503 ± 0.0008	0.16	0.67
1	1.0003 ± 0.005	0.56	0.03	0.99 ± 0.0007	0.08	0.08
5	5.009 ± 0.013	0.26	0.2	5.004 ± 0.008	0.16	0.084
15	14.99 ± 0.06	0.42	0.06	14.98 ± 0.02	0.13	0.13
25	25.04 ± 0.09	0.35	0.16	24.99 ± 0.07	0.27	0.005

#### Accuracy

Method accuracy or trueness describes the closeness of observed quantities to their theoretical values and was evaluated using the ‘standard additions’ method. The selected additions of LNG used in this study were 25%, 50%, 100% and 200%, and the accuracies (based on measured mass compared to theoretical values) were 102.8%, 102.8%, 102.2% and 104.9% respectively, with relative errors of less than 5% (Table 3).

**Table 3:** HPLC–UV method accuracy using the standard additions method. A known volume of an LNG standard solution was added to 100 ng of LNG (100 µL of the 1 µg/mL calibration standard) to achieve a 25, 50, 100 or 200% increase in the LNG mass on column. The measured masses were then compared to the theoretical values to determine the accuracy by %RSD and %RE

**LNG mass added (%)**	**Total theoretical mass (ng)**	**Measured mass ± SD (ng)**	**RSD (%)**	**RE (%)**
25	125	128.5 ± 3.53	2.75	2.79
50	150	154.16 ± 1.51	0.97	2.77
100	200	204.47 ± 0.93	0.45	2.23
200	300	314 ± 4.86	1.54	4.87

### Development of an LLE procedure to isolate LNG from human skin homogenate medium

**Fig. 3:**
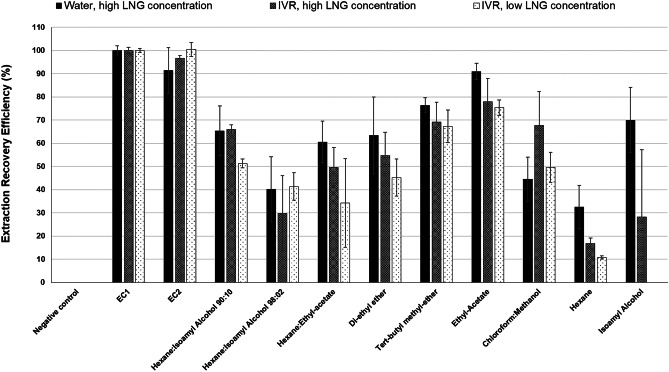
The extraction recovery efficiencies of different organic solvents from two simple media (water and IVR medium) using a ‘high’ (1.6 µg/mL) or ‘low’ (0.4 µg/mL) spiked LNG concentration. Extraction recovery efficiencies were calculated in comparison to an untreated extraction control (EC1) which was not subject to any of the extraction steps. Data shown are the means ± SD. For water samples; *n* = 6. For IVR samples at high concentration; *n* = 3 except for tert-butyl methyl-ether and isoamyl alcohol where *n* = 2, while at low concentration; *n* = 2 each except for isoamyl alcohol where *n* = 0

The use of SH medium for drug release studies will require a drug extraction and recovery method that separates the released analyte from the diverse biological interferent. In this study, a novel two-step liquid–liquid extraction (LLE) procedure for the efficient recovery of LNG from a dermal matrix is reported. The LLE procedure was developed in three stages. In Stage 1, several organic solvents were examined as means to extract LNG from a simple aqueous solution spiked with a ‘high’ (1.6 µg/mL) LNG concentration. The extraction recoveries ranged between 32.5 ± 9.5% (*n *= 6) for hexane and 91 ± 3.5% (*n *= 6) for ethyl acetate, which was the best performing organic solvent. Similar results were observed with the IVR samples spiked with high (1.6 µg/mL) and ‘low’ (0.4 µg/mL) LNG concentrations where ethyl acetate achieved the highest recovery efficiencies, 77.94 ± 10.03% (*n* = 3) and 75.3 ± 3.38% (*n* = 2), respectively. Figure 3 summarises the extraction recovery efficiencies achieved using multiple organic phases in comparison to the positive extraction control, EC1.

**Fig. 4:**
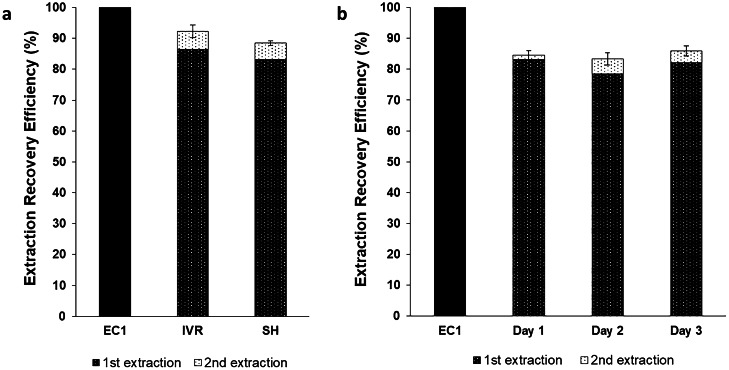
Optimisation and validation of a two-step LLE (liquid–liquid extraction) procedure to recover LNG from IVR or SH media. (**a)** The extraction recovery efficiencies of LNG from spiked IVR and SH media samples (19 µg/mL of LNG) compared to the untreated extraction control (EC1). The two-step extraction procedure used 500 and 150 µL of ethyl acetate, respectively. Data for IVR and SH media is the mean average ± SD (*n* = 3). (**b)** Repeated analysis of the extraction recovery efficiencies of LNG from spiked SH medium (3.6 µg/mL of LNG) compared to EC1. Experiments were repeated daily, in triplicate, for 3 consecutive days and used the optimised two-step extraction procedure (Fig. 1). Data for day 1, day 2 and day 3 is the mean average ± SD (*n* = 3)

After selecting ethyl acetate as the preferred extraction solvent, a two-step extraction procedure was evaluated (Stage 2) to optimise LNG recovery from the more complex biological SH medium whilst also reducing the volumes of organic solvent used in the process (35% reduction). In this two-step procedure, LNG recovery increased between the 1st and 2nd extractions, achieving a combined recovery efficiency of 92.5 ± 2% in IVR (*n* = 3) and 88.5 ± 1% in SH (*n* = 3) (Fig. 4a). The two-step extraction method was further validated over 3 consecutive days (Stage-3) to provide mean intra- and inter-day recovery efficiencies (Fig. 4b; Supplementary Table 3). The final extraction efficiency of LNG from SH medium using the optimised two-step extraction procedure and increased experimental replicates was 84.61 ± 1.64% (*n* = 9).

LNG has previously been isolated and quantified from various biological samples using similar LLE protocols: the volume of extracted sample, number of extraction cycles and organic solvent being the main variables. Sample volume depends on the nature of the sample, e.g. 5 mL for breastfeeding milk samples [26] and between 100 µL and 1 mL [31–33, 36, 40] for plasma and serum samples. The current study used 500 µL of SH medium for LLE. Whilst extractions from plasma/serum samples have generally adopted one-step LLE, the employment of a two-step protocol was reported for breastfeeding milk samples, which was complemented with RIA to yield an 82% extraction recovery efficiency [26]. The second extraction step used in this study enhanced the total extraction efficiency of LNG from SH medium by a further 3.4 ± 1.75% (*n* = 9). Although further extraction cycles could incrementally improve LNG recovery, this must be balanced against the additional time and volume of solvent required. With respect to the extraction solvent, ethyl-acetate was found to achieve the highest recovery efficiencies. Our results contrast with data obtained using human plasma for example, where diethyl ether was a more effective organic solvent for extracting LNG than ethyl acetate [41].

The extraction efficiency reported in this study is generally comparable to published extraction efficiencies for LNG from human plasma, e.g. 85% [35] and 84–90% [33], although one published study achieved a recovery efficiency of 99.5% [32]. Skin is a highly complex and stratified biological matrix containing collagen fibres, a variety of cell populations and UV-absorbing molecules [58]. Therefore, the analyses of molecules extracted from skin or cutaneous suspensions are particularly challenging, given the necessity for sample pre-treatment to eliminate cutaneous interferent and the requirement to achieve acceptable chromatographic separation.

### Evaluation of LNG temporal release in in vitro release medium and ex vivo human skin homogenate medium using a commercially available sustained release formulation

**Fig. 5:**
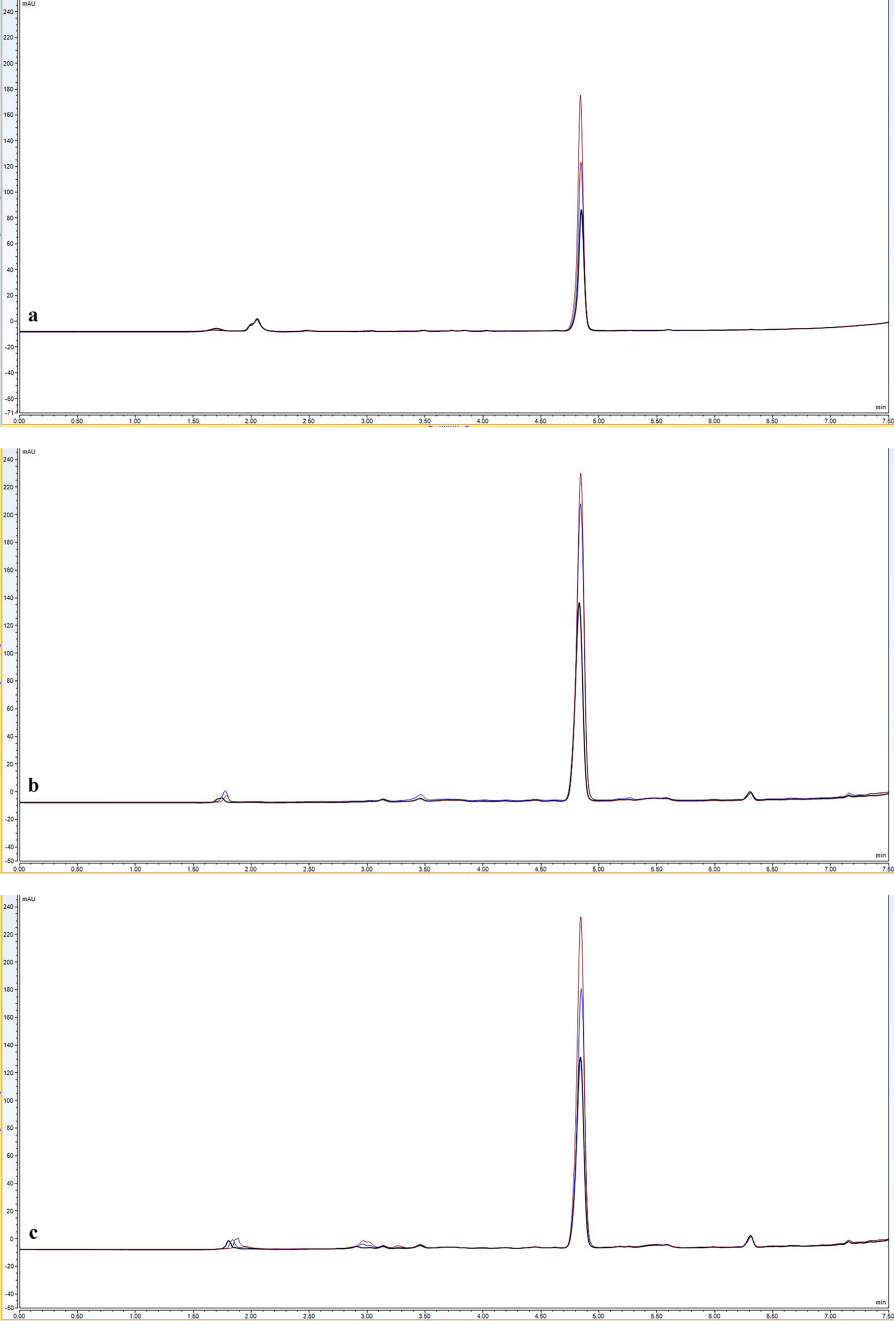
Illustrative overlaid HPLC–UV chromatograms of LNG released from Jadelle® implants in IVR (**a**, **b**) and SH (**c**) media. Samples of IVR (*n* = 1) and SH media (*n* = 1) were collected at days 7 (black), 14 (blue) and 28 (brown). LNG in IVR samples (**a)** was directly assayed by HPLC using the optimised method (“Optimised HPLC–UV method”). Further IVR samples (**b)** were subjected to an extraction step to illustrate the efficiency of the extraction procedure. In SH samples, (**c)** LNG was also recovered using the optimised extraction procedure (“LLE of LNG from human skin homogenate medium”) prior to analysis

Jadelle® subcutaneous implants (also known as Norplant II) are a sustained release contraceptive product containing 150 mg of crystalline LNG mixed with dimethyl-siloxane/methylvinyl-siloxane copolymer and covered with a rate-limiting silicone membrane. These implants are designed to be surgically inserted into the sub-cutaneous space, typically on the upper arm, and slowly release LNG to provide contraceptive effect up to 5 years [59]. In this study, Jadelle® implants were used to demonstrate whether the supplemented release models (IVR and SH media) and accompanying extraction and analytical methodologies permit the characterisation of LNG release over prolonged periods (Fig. 5).

The chromatograms demonstrate that LNG released from Jadelle® implants maintained a similar retention time to the calibration standards (~ 4.8 min) at all time points over 28 days and in both IVR and SH media, with minimal background interference. As expected, the peak area attributed to LNG increased with time, with the greatest release observed within the first 7 days, due to the acknowledged initial higher ‘burst’ release profile of Jadelle® [60] (Fig. 5). The chromatograms suggest that there is a small amount of sample contamination from materials emanating from the Jadelle® implant (apparent in all samples at a shorter retention time than the LNG peak) and from the extraction protocol (apparent in samples subjected to the extraction step at a longer retention time than the LNG peak). Whilst any contaminants from the complex SH medium did not appear to significantly interfere with the analytical method (Fig. 5c), it would be important to include a negative control sample of SH medium when conducting quantitative drug release studies to account for potential background interference at the LNG retention time.

The peak ratios of LNG released in IVR were comparable both without (Fig. 5a) and with (Fig. 5b) the solvent extraction step, indicating that the extraction process does not adversely affect LNG recovery or analysis; the heights and AUCs of these peaks are different as the sample volumes used to load the column are not the same. Equivalent sample volumes were however used in samples derived from LNG extracted from IVR medium (Fig. 5b) and the more complex biological SH medium (Fig. 5c). The peak heights and AUCs derived from these samples were extremely similar, indicating comparable LNG release characteristics in both media. This provides preliminary evidence that IVR medium alone, with the accompanying analytical methodology, could be valuable in rapid, high-throughput pre-clinical screening and optimisation of innovative intradermal sustained release LNG formulations. Further validation work is of course required to ensure that these models are predictive; LNG release studies should be performed to reflect the proposed clinical duration of action and parallel studies should be performed in animal models to determine in vitro-in vivo correlation.

## Conclusion

In vitro (IVR medium) and ex vivo (SH medium) laboratory models of human skin and a robust, rapid, sensitive analytical methodology have been developed, optimised and characterised to determine LNG release from intradermally administered sustained delivery dosage forms. The release models, even when supplemented with a homogenate of excised human skin to improve biological relevance, permit the characterisation of sustained drug release over weeks and months. The analytical method, employing a simple two-step liquid–liquid extraction procedure followed by reversed-phase HPLC–UV, is able to accurately quantify nanogram per mL levels of LNG and addresses the two main challenges of drug extraction from biological models — recovery of the drug and separation from the diverse biological interferent. In vivo data is now required to further exemplify the utility of the in vitro/ex vivo models and methodology to evaluate emerging intradermal controlled release contraceptive products, such as LNG-loaded biodegradable MNs [13, 14].

## Supplementary Information

Below is the link to the electronic supplementary material.

Supplementary file1 (JPG 70 KB)

Supplementary file2 (JPG 74 KB)

Supplementary file3 (TIF 128 KB)

Supplementary file4 (DOCX 15 KB)

## Data Availability

Access to data will be granted from the authors on reasonable request.

## Abbreviations

ACN: Acetonitrile
AUC: Area under curve
EC: Extraction control
DMEM: Dulbecco’s modified Eagle’s medium
FDA: US Food Drug Administration
ICH: International Council for Harmonisation
IVR: In vitro release medium
LARC: Long-acting reversible contraceptive
LC-MS: Liquid chromatography-mass spectrometry
LLE: Liquid-liquid extraction
LNG: Levonorgestrel
LOD: Limit of detection
LOQ: Limit of quantification
MN: Microneedle
PBS: Phosphate-buffered saline
PPT: Protein precipitation
RE: Relative error
RIA: Radioimmunoassay
RP: Reversed-phase
HPLC-UV: High-performance liquid chromatography-Ultraviolet
RSD: Relative standard deviation
RT: Retention time
SDS: Sodium dodecyl sulphate
SH: Skin homogenate
S/N: Signal-to-noise
SPE: Solid phase extraction
