# Supplementary figures and images for: Models and methods to characterise levonorgestrel release from intradermally administered contraceptives
Source: Drug Deliv Transl Res. 2021 Dec 3;12(2):335–49. doi: 10.1007/s13346-021-01091-5 (PMC8724103; doi:10.1007/s13346-021-01091-5)

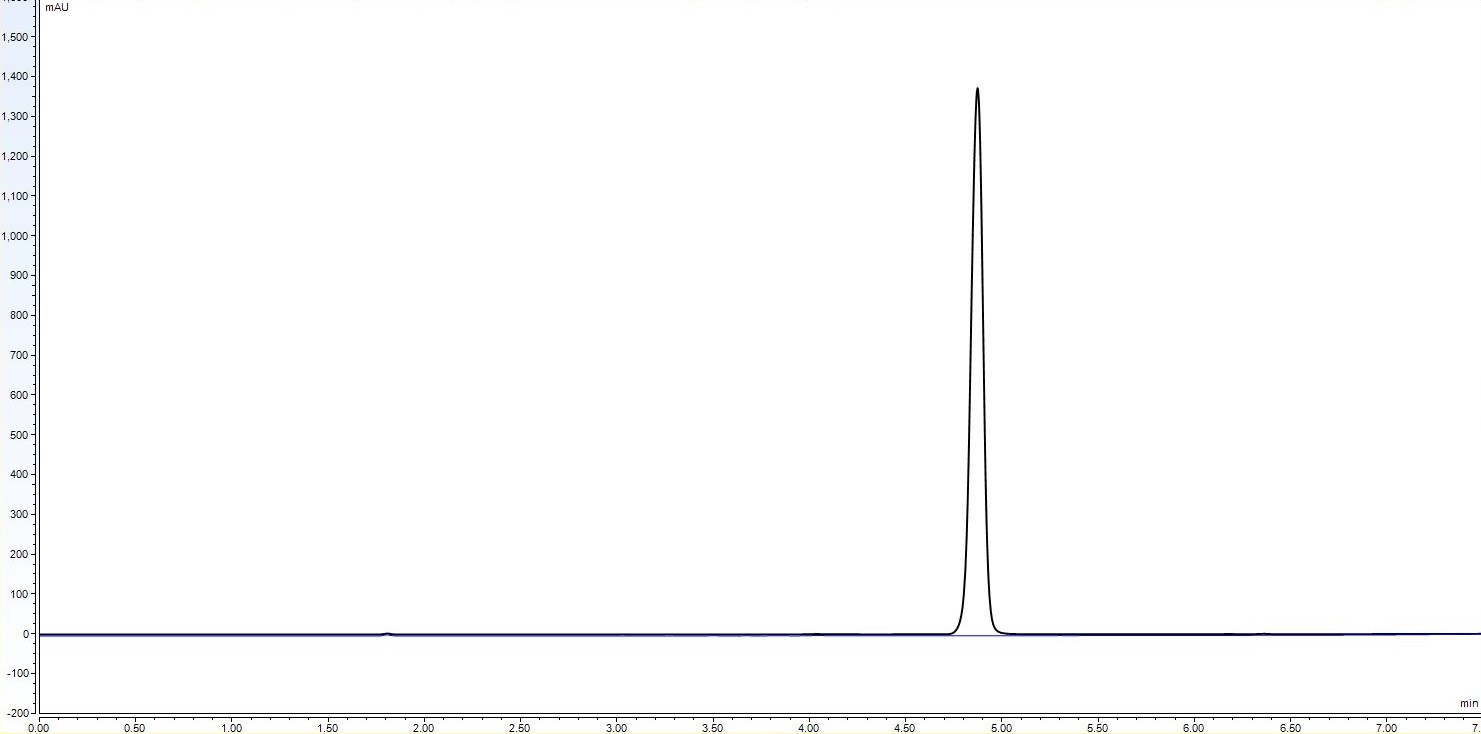

Supplement: Supplementary file 1 — Supplementary file1 (JPG 70 KB) [file 13346_2021_1091_MOESM1_ESM.jpg]

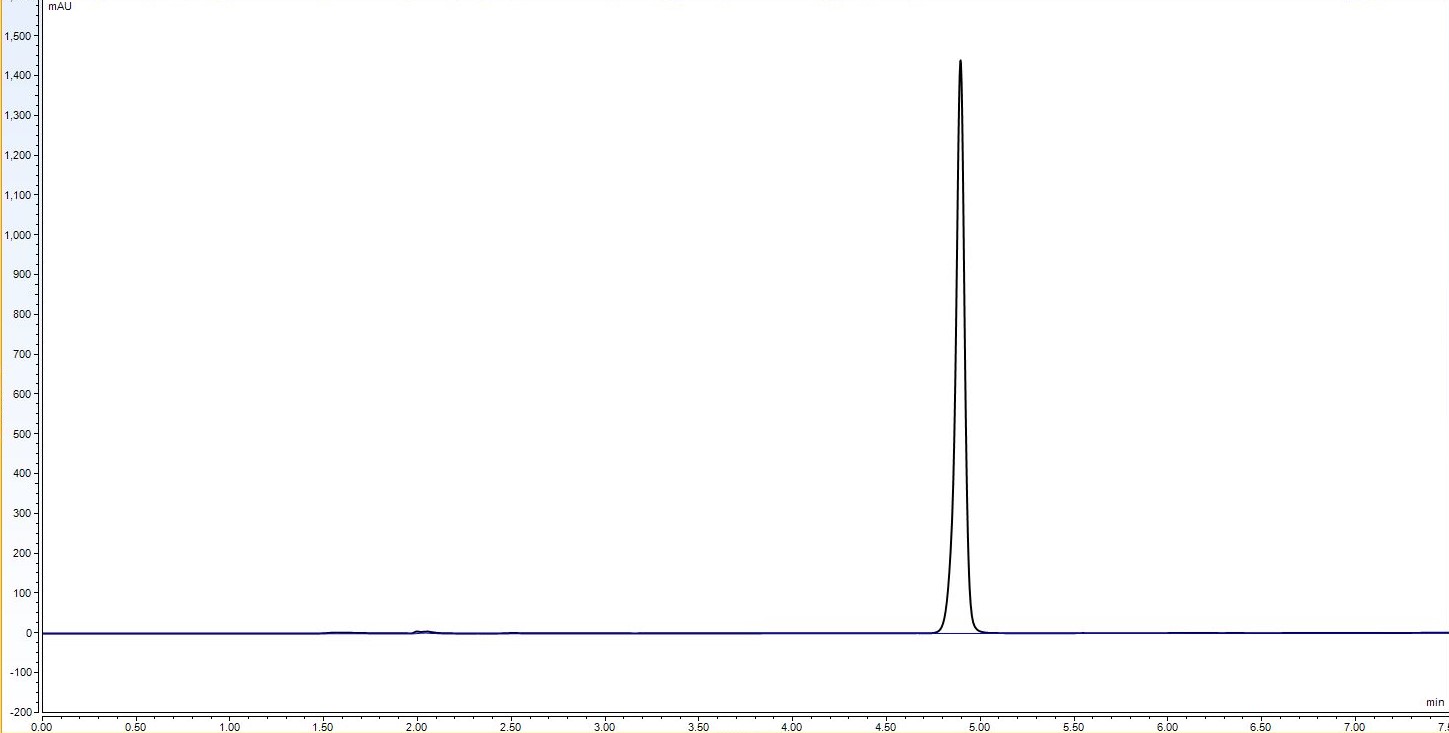

Supplement: Supplementary file 2 — Supplementary file2 (JPG 74 KB) [file 13346_2021_1091_MOESM2_ESM.jpg]

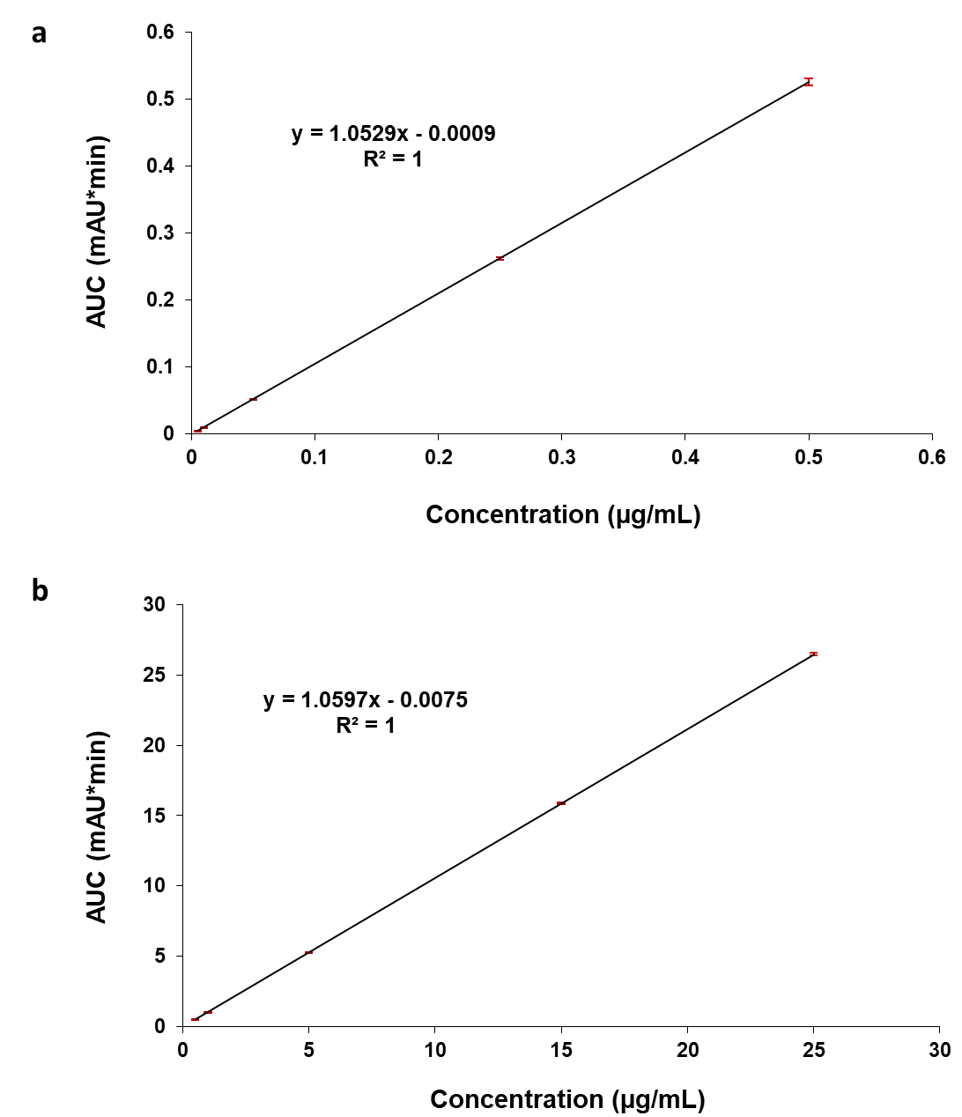

Supplement: Supplementary file 3 — Supplementary file3 (TIF 128 KB) [file 13346_2021_1091_MOESM3_ESM.tif]
