# Supplementary material for: Models and methods to characterise levonorgestrel release from intradermally administered contraceptives
Source: Drug Deliv Transl Res. 2021 Dec 3;12(2):335–49. doi: 10.1007/s13346-021-01091-5 (PMC8724103; doi:10.1007/s13346-021-01091-5)
Supplement: Supplementary file 4 — Supplementary file4 (DOCX 15 KB) [file 13346_2021_1091_MOESM4_ESM.docx]

**Supplementary Table 1 Optimisation of HPLC-UV assay parameters for LNG quantification**

| **Condition** | **Run time (min)** | **Elution mode** | **Mobile phase  (ACN:H_2_O)** | **Column temperature (°C)** | **Detection wavelength (nm)** | **Flow rate (mL/min)** | **Retention time (min)** |
| --- | --- | --- | --- | --- | --- | --- | --- |
| 1 | 20 | Isocratic | 50:50 | 25 | 236 | 1 | 9.567 |
| 2 | 12 | Isocratic | 55:45 | 25 | 236 | 1 | 7.382 |
| 3 | 12 | Isocratic | 60:40 | 25 | 236 | 1 | 5.973 |
| 4 | 12 | Isocratic | 65:35 | 25 | 236 | 1 | 5.055 |
| 5 | 12 | Isocratic | 70:30 | 25 | 236 | 1 | 4.423 |
| 6 | 12 | Isocratic | 75:25 | 25 | 236 | 1 | 3.978 |
| 7 | 12 | Isocratic | 80:20 | 25 | 236 | 1 | 3.658 |
| 8 | 12 | Isocratic | 85:15 | 25 | 236 | 1 | 3.435 |
| 9 | 12 | Isocratic | 90:10 | 25 | 236 | 1 | 3.283 |
| 10 | 12 | Isocratic | 95:05 | 25 | 236 | 1 | 3.193 |
| 11 | 12 | Isocratic | 100:00 | 25 | 236 | 1 | 3.223 |
| 12 | 12 | Gradient | 60:40 to 90:10 at 4.4 to 5.5 min | 25 | 236 | 1 | 5.105 |
| 13 | 12 | Gradient | 60:40 to 95:05 at 4.4 to 5.5 min | 25 | 236 | 1 | 5.033 |
| 14 | 12 | Gradient | 60:40 to 100:00 at 4.4 to 5.5 min | 25 | 236 | 1 | 4.965 |
| 15 | 20 | Isocratic | 60:40 | 25 | 236 | 0.5 | 12.968 |
| 16 | 12 | Isocratic | 60:40 | 25 | 236 | 0.75 | 8.205 |
| 17 | 12 | Isocratic | 60:40 | 25 | 236 | 1.25 | 4.792 |
| 18 | 12 | Isocratic | 60:40 | 25 | 225 | 1 | 5.967 |
| 19 | 12 | Isocratic | 60:40 | 25 | 230 | 1 | 5.975 |
| 20 | 12 | Isocratic | 60:40 | 25 | 240 | 1 | 5.967 |
| 21 | 12 | Isocratic | 60:40 | 25 | 234 | 1 | 5.97 |
| 22 | 12 | Isocratic | 60:40 | 25 | 238 | 1 | 5.975 |
| 23 | 12 | Isocratic | 60:40 | 25 | 242 | 1 | 5.97 |
| 24 | 12 | Isocratic | 60:40 | 25 | 244 | 1 | 5.973 |
| 25 | 12 | Isocratic | 60:40 | 25 | 246 | 1 | 5.975 |
| 26 | 12 | Isocratic | 60:40 | 25 | 248 | 1 | 5.978 |
| 27 | 12 | Gradient | 60:40 to 100:00 at 4.4 to 5.5 min | 25 | 236 | 1 | 4.98 |
| 28 | 12 | Gradient | 60:40 to 100:00 at 4.4 to 5.5 min | 30 | 236 | 1 | 4.913 |
| 29 | 12 | Gradient | 60:40 to 100:00 at 4.4 to 5.5 min | 35 | 236 | 1 | 4.843 |
| 30 | 12 | Gradient | 60:40 to 100:00 at 4.4 to 5.5 min | 40 | 236 | 1 | 4.772 |
| 31 | 12 | Gradient | 60:40 to 100:00 at 4.4 to 5.5 min | 45 | 236 | 1 | 4.693 |
| 32 | 12 | Gradient | 60:40 to 100:00 at 4.4 to 5.5 min | 50 | 236 | 1 | 4.612 |
| 33 | 7.5 | Gradient | 60:40 to 100:00 at 4.4 to 5 min | 37 | 244 | 1 | 4.825 |

**Supplementary Table 2 Evaluation of HPLC assay sensitivity using signal/noise method.**

The ratio S/N was calculated at three minimal concentrations and the mean values were used to determine LOD and LOQ

| **LNG concentration**  **(µg/mL)** | **Replicate #** | **H (cm)** | **H (cm)** | **S/N = 2H/h**  **Average** | |
| --- | --- | --- | --- | --- | --- |
| 0.001 | 1 | 0.9 | 0.5 | 3.6 | 3.6 > 3×S/N (LOD) |
|  | 2 | 1 | 0.5 | 4 |  |
|  | 3 | 0.8 | 0.5 | 3.2 |  |
| 0.0025 | 1 | 2.2 | 0.55 | 8 | 8 |
|  | 2 | 2.4 | 0.6 | 8 |  |
|  | 3 | 2 | 0.5 | 8 |  |
| 0.005 | 1 | 3.8 | 0.65 | 11.69 | 12.56 > 10×S/N (LOQ) |
|  | 2 | 4.1 | 0.6 | 13.66 |  |
|  | 3 | 3.7 | 0.6 | 12.33 |  |

**Supplementary Table 3 Optimisation of the extraction procedure over 3 consecutive days.** The data shown are the mean values of 3 extracted samples ± SD

| **LNG concentration (µg/mL)** | **Extraction Recovery Efficiency %** | | | | | | |
| --- | --- | --- | --- | --- | --- | --- | --- |
|  | **Intra-day (n=3 on each day)** | | | | | **Inter-day on 3 consecutive days (n=9)** | |
|  | **Day #** | **Mean Efficiency of the 1^st^ extraction** | **Mean Efficiency of the 2^nd^ extraction** | **Mean Total Efficiency** | **± SD** | **Mean Total Efficiency** | **± SD** |
| 3.6 | 1 | 83.1 | 1.45 | 84.55 | 1.4 | 84.61 | 1.64 |
|  | 2 | 78.45 | 4.86 | 83.32 | 1.9 |  |  |
|  | 3 | 82.07 | 3.88 | 85.96 | 1.6 |  |  |
